# A Biocompatible and Biodegradable Protein Hydrogel with Green and Red Autofluorescence: Preparation, Characterization and *In Vivo* Biodegradation Tracking and Modeling

**DOI:** 10.1038/srep19370

**Published:** 2016-01-27

**Authors:** Xiaoyu Ma, Xiangcheng Sun, Derek Hargrove, Jun Chen, Donghui Song, Qiuchen Dong, Xiuling Lu, Tai-Hsi Fan, Youjun Fu, Yu Lei

**Affiliations:** 1 Department of Biomedical Engineering, University of Connecticut, Storrs, CT 06269, USA; 2 Department of Chemical and Biomolecular Engineering, University of Connecticut, Storrs, CT 06269, USA; 3 Department of Pharmaceutical Sciences, University of Connecticut, Storrs, CT 06269, USA; 4 Department of Mechanical Engineering, University of Connecticut, Storrs, CT 06269, USA; 5 Department of Chemistry, University of Connecticut, Storrs, CT 06269, USA

## Abstract

Because of its good biocompatibility and biodegradability, albumins such as bovine serum albumin (BSA) and human serum albumin (HSA) have found a wide range of biomedical applications. Herein, we report that glutaraldehyde cross-linked BSA (or HSA) forms a novel fluorescent biological hydrogel, exhibiting new green and red autofluorescence *in vitro* and *in vivo* without the use of any additional fluorescent labels. UV-vis spectra studies, in conjunction with the fluorescence spectra studies including emission, excitation and synchronous scans, indicated that three classes of fluorescent compounds are presumably formed during the gelation process. SEM, FTIR and mechanical tests were further employed to investigate the morphology, the specific chemical structures and the mechanical strength of the as-prepared autofluorescent hydrogel, respectively. Its biocompatibility and biodegradability were also demonstrated through extensive *in vitro* and *in vivo* studies. More interestingly, the strong red autofluorescence of the as-prepared hydrogel allows for conveniently and non-invasively tracking and modeling its *in vivo* degradation based on the time-dependent fluorescent images of mice. A mathematical model was proposed and was in good agreement with the experimental results. The developed facile strategy to prepare novel biocompatible and biodegradable autofluorescent protein hydrogels could significantly expand the scope of protein hydrogels in biomedical applications.

Cross-linked hydrogel networks, imbibing a large volume of water and maintaining three dimensional structure^1^,^2^, have been widely investigated for biomedical applications ranging from drug delivery to tissue engineering^2^,^3^,^4^. Many materials, including natural proteins and synthetic polymers, have been used to prepare hydrogels^5^. Among different hydrogels that have been reported, protein hydrogels are of great interest because there is only a slight frictional irritation upon their implantation in tissue, due to their softness and good biocompatibility^6^. In addition, protein hydrogels generally exhibit a highly porous structure, relatively good hydrophilicity, biodegradability, and also enhanced biocompatibility, which could greatly suppress the foreign body reaction for *in vivo* applications^7^. Nowadays, protein-based hydrogels containing collagen and gelatin are most widely synthesized using glutaraldehyde or water-soluble carbodiimides^8^ as the cross-linkers. Beyond the aforementioned proteins and their based hydrogels, bovine serum albumin (BSA) and human serum albumin (HSA) and their based hydrogels have also found plenty of applications owing to its low cost, stability, unusual ligand-binding properties and high solubility^9^,^10^. BSA (or HSA) has two tryptophan residues that are responsible for its intrinsic fluorescence (excitation/emission of 279 nm/348 nm), however, the excitation and emission in the UV range significantly diminish any *in vivo* application of using their intrinsic fluorescence. Glutaraldehyde (GA), a linear 5-carbon dialdehyde with a formula of CH_2_(CH_2_CHO)_2,_ is an apparent, colorless, pungent oily liquid that is soluble in all proportions in water and alcohol, as well as in organic solvents^11^. Glutaraldehyde is a widely used cross-linker because of its low cost, high reactivity, and good efficiency to react with the amine groups to generate thermal and chemical stable crosslinks compared with other aldehydes^12^. Although several functional groups in protein can react with glutaraldehyde in aqueous solution^11^, including amine, thiol, imidazole, etc^13^., GA possesses high reactivity with lysine residues (ϵ-amino groups)^11^. Therefore, GA is predominantly reacted with the ϵ-amino groups of lysine in protein to form major intermolecular cross-linkages^13^. Although GA has been previously used in BSA nanoparticle preparation^14^, there is no report on its use to prepare hydrogels with green and red autofluorescence.

In the past few decades, fluorescent hydrogel has attracted more and more research interests due to its practical application as a convenient tracer in biomedical engineering^15^,^16^. Many fluorescence-based chemical, physical and biological hydrogels have been developed for glucose sensing^17^,^18^, insulin-sensing^19^, temperature-responsive sensing^20^, as well as for tracking *in vitro* and *in vivo* degradation of hydrogels^15^,^16^. However, to synthesize those fluorescent hydrogels, fluorophores are either chemically or physically immobilized in the hydrogel matrix, endowing the fluorescence property to the hydrogels. Although different covalent conjugation and physical entrapment methods have been developed to anchor fluorophores, their applicability is greatly limited because of the complexity of synthetic routes, the potential leakage and photobleaching of fluorophores, the influence of fluorescent labels on the degradation pattern, as well as the concerns of biocompatibility and biodegradability of those fluorophores. Ideally, an autofluorescent protein hydrogel with a suitable excitation/emission wavelength is highly favorable in this regard.

In this study, we report a unique glutaraldehyde cross-linked albumin (BSA or HSA) hydrogel which exhibits strong green and red autofluorescence over a large excitation wavelength range. To the best of our knowledge, such unique green and red fluorescence of BSA (or HSA) hydrogel has not been reported in literature. The absorption and fluorescence properties of the as-prepared autofluorescent hydrogel were extensively investigated through UV-vis spectra and the fluorescence spectra including emission, excitation and synchronous scans. After lyophilization to remove unreacted glutaraldehyde, Scanning Electron Microscopy (SEM) and Fourier Transform Infrared Spectroscopy (FTIR) were further employed to investigate the surface morphology and the specific chemical structures of the lyophilized hydrogel sample, respectively. The hydrogel also exhibited high toughness according to the compression and tensile tests. Finally its biocompatibility and biodegradability were demonstrated through extensive *in vitro* and *in vivo* studies. More interestingly, the *in vivo* degradation of autofluorescent hydrogel can be non-invasively tracked using fluorescence images, which provided a convenient way to model *in vivo* biodegradation of the protein hydrogel from a new perspective. The degradation/diffusion trends predicted by the proposed mathematical model were in good agreement with the time-dependent fluorescence images of mice. These results also indicate that the newly formed fluorophores in the autofluorescent BSA hydrogel are stable and not degraded after protein degration until they are finally absorbed into circulation. An attempt to identify the chemical structures of newly formed fluorophores in proteinase K digested hydrogel sample using LC-MS turns out to be challenging due to the complexity of GA cross-linked BSA hydrogel. The developed facile strategy to prepare a unique autofluorescent protein hydrogel could significantly expand the scope of protein hydrogels in biomedical applications.

## Results

### Gelation of BSA using glutaraldehyde

**Figure 1:**
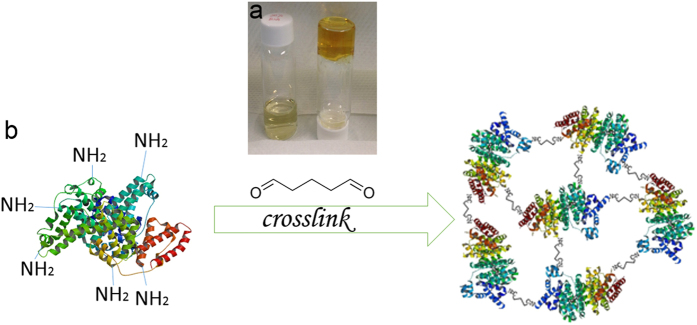
(**a**) The right vial contains BSA hydrogel after GA cross-linking, while the left vial contains 20% BSA solution as a comparison. (**b**) The schematic of GA-cross-linked BSA hydrogel (not in scale).

As reported, glutaraldehyde predominantly crosslinks ϵ-amino groups of lysine within BSA^13^. Since the concentration of BSA solution used in this study is very high, the intermolecular cross-linking process generates extensive 3D networks, thus forming a protein hydrogel. To investigate the gelation time, we employed a test tube inversion method reported in literature^21^. The time when the polymer solution stops flowing out of inverted tubes was recorded at 25 °C. Briefly, BSA was first dissolved in 0.01 M pH 7.4 PBS buffer solution and then glutaraldehyde was added into BSA solution as the cross-linker with the final concentration of 20% BSA and 1% GA. After adding glutaraldehyde, the clear dark yellow protein hydrogel which does not flow upon inversion, was obtained within 30–45 seconds (Fig. 1a). Differential Scanning Calorimetry (DSC) and Thermogravimetric Analysis (TGA) techniques were employed to study the thermal properties (e.g., melting point and decomposition temperature) of the cross-linked BSA hydrogel and the results (Figure S2) and discussion are presented in supplemental materials. The schematic representation of the GA cross-linked BSA hydrogel was presented in Fig. 1b.

### UV-vis study of cross-linked BSA hydrogel

**Figure 2:**
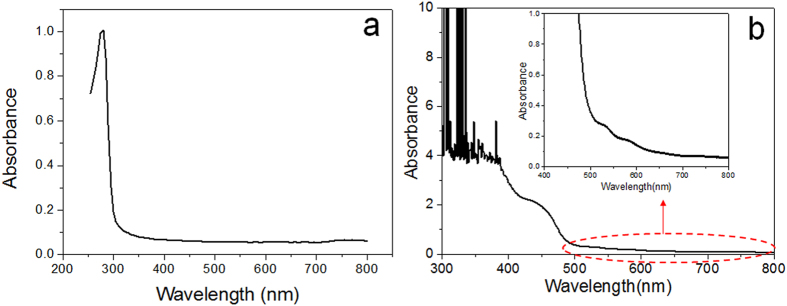
UV-vis spectra of BSA solution (**a**) and GA cross-linked BSA hydrogel (**b**), respectively.

According to Fig. 1, the cross-linking process by GA not only forms BSA hydrogel, but also probably produces new colorful compounds, endowing the obvious color difference between BSA hydrogel and BSA solution. Therefore, a UV-vis spectrum was employed as a useful probe for initial composition analysis. Spectral analysis shows that BSA in aqueous solution has a sharp absorption peak at λ = 279 nm (Fig. 2a), which is typical for BSA proteins. To investigate the newly formed colorful compounds in the cross-linked BSA hydrogel, UV-vis spectrum scanning from 300 nm to 800 nm was utilized (Fig. 2b). Compared to the UV-vis spectrum of BSA solution, GA cross-linked BSA hydrogel showed three new broad peaks centered around 460 nm, 530 nm and 580 nm, respectively. This suggests that there might have three classes of compounds formed during BSA cross-linking by GA, which led to further investigation into the fluorescence properties of the GA cross-linked BSA hydrogel.

### Fluorescent images of cross-linked BSA hydrogel

**Figure 3:**
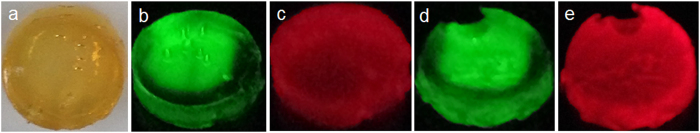
**(a)** BSA hydrogel under normal white light;(**b**) BSA hydrogel image (excited at 470 nm) is collected using a λ = 530 nm optical filter; (**c**) BSA hydrogel image (excited at 595 nm) is collected using a λ = 630 nm optical filter. (**d**) HSA hydrogel image (excited at 470 nm) is collected using a λ = 530 nm optical filter. (**e**) HAS hydrogel image (excited at 595 nm) is collected using a λ = 630nm optical filter.

BSA, a well-known protein fluorophore, has a strong emission at ~348 nm when it is excited at λ = 279 nm. However, according to the UV-vis spectrum of BSA hydrogel in Fig. 2b, BSA hydrogel might exhibit fluorescence when excited in a broad wavelength range. To test the hyphothesis, different optical filters (Ex = 470 nm and Em = 530 nm; Ex = 595 nm and Em = 630 nm) were first used to acquire fluorescent images of the cross-linked BSA hydrogel. Interestingly and unexpectedly, cross-linked BSA hydrogel exhibits strong green and red fluorescence, respectively (Fig. 3). Under white light, the as-prepared BSA hydrogel is dark yellow color (Fig. 3a). However, when BSA hydrogel was excited at a wavelength of λ = 470 nm and λ = 595 nm, respectively, the BSA hydrogel exhibited a strong green fluorescence (Fig. 3b) and red fluorescence (Fig. 3c) when the images were collected using an optical bandpass filter with λ = 530 nm and λ = 630 nm, respectively. In conjunction with the results from UV-vis spectra, fluorescence images of BSA hydrogel suggested that there indeed have new classes of fluorescent molecules formed in the cross-linked BSA hydrogel. Besides BSA, human serum albumin (HSA) can also be cross-linked to show strong green and red autofluorescence (Fig. 3d–e). For subsequent studies, only BSA cross-linked hydrogel is used due to its low cost compared to HSA.

### Fluorescence spectra study of cross-linked BSA hydrogel

**Figure 4:**
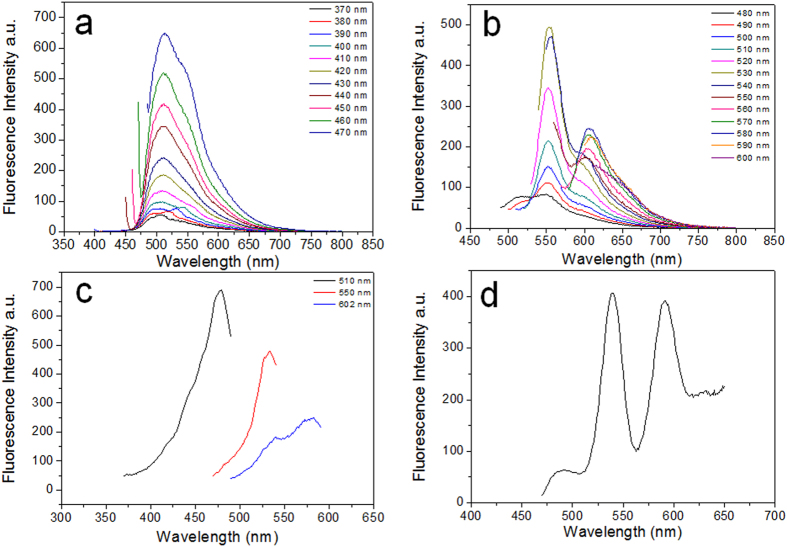
(**a**) and (**b**) Emission scans when excited between λ = 370 nm and λ = 600 nm, split into two figures for the sake of clear display. (**c**) Three Excitation scans with the Emission wavelength at λ = 510 nm, 550 nm, 602 nm, respectively. (**d**) Synchronous scan.

Emission spectra of BSA hydrogel is further recorded with a fluorometer. A total of 24 individual emission spectra at sequential 10 nm increments of excitation wavelength were recorded. According to Fig. 4a,b, there existed obvious emission peaks in the green fluorescence wavelength range when it was excited at a wavelength between λ = 370 nm and λ = 530 nm. Moreover, it shows that the emission peak shifts slightly from λ = 510 nm to λ = 550 nm over the same excitation range. When excited at a wavelength between 520 nm and 590 nm, a broad emission peak centered around λ = 602 nm appeared, which is attributed to the the observed red fluorescence of BSA and HSA hydrogels. Excitation scan was also conducted and the results (Fig. 4c) suggest that the proper excitation wavelengths for the three emission peaks (510, 550 and 602 nm) are 478, 530, and 580 nm, respectively. Moreover, the synchronous scan in Fig. 4d also shows distinct emission peaks which may correspond to three classes of fluorescent compounds in the cross-linked BSA hydrogel. These observations are not only in good agreement with the results suggested by UV-vis spectra in Fig. 2, but also corroborate the strong green and red antofluorescence observed in Fig. 3.

### SEM and FTIR characterization of cross-linked BSA hydrogel

**Figure 5:**
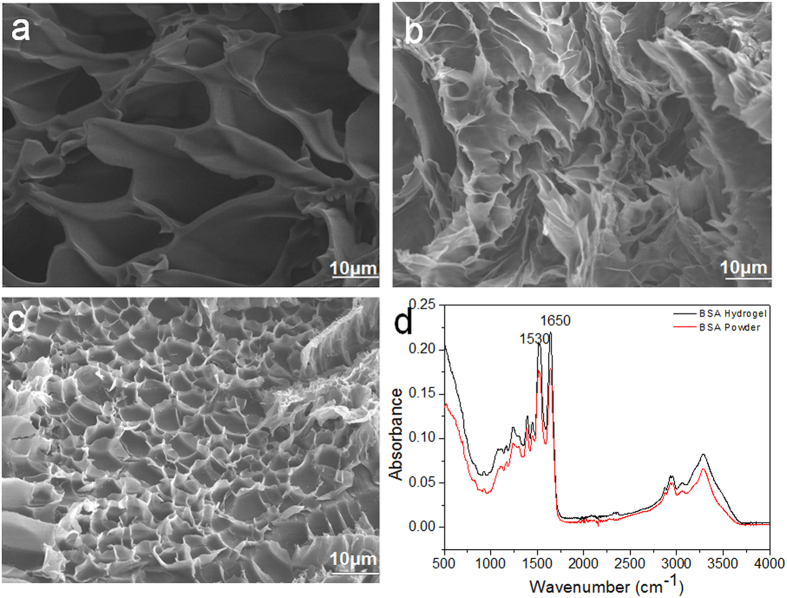
(**a**) The SEM image of 10% BSA hydrogel; (**b**) The SEM image of 20% BSA hydrogel; (**c**) The SEM image of 30% BSA hydrogel; (**d**) The FTIR spectra of BSA hydrogel and BSA powder, respectively.

To characterize the morphology and specific functional groups of the cross-linked BSA hydrogel, SEM and FTIR were first employed. The cross-sectional interior structures of flash-frozen and lyophilized BSA hydrogels with different BSA concentrations (10%, 20% and 30%) are shown in Fig. 5. All three BSA hydrogels exhibited a highly porous honeycomb-like structure with an irregular shape in which the three-dimensional, interconnected macropores represent water-filled areas, while the pore size features a BSA concentration-dependent property. In general, an increase in the concentration of BSA results in a smaller pore size, as expected. Specifically, the hydrogel with 10% BSA was characterized with an average pore size of 20 μm. The 20% BSA hydrogel exhibited a pore size of 10 μm, while the 30% BSA hydrogel exhibited a very dense structure with an average pore size of 5 μm. For the FTIR study, a freeze-dried 20% BSA hydrogel sample and the native BSA powder were used. As shown in Fig. 5d, both BSA powder and BSA hydrogel showed nearly the same FTIR spectrum with a fingerprint region at 1650 and 1530 cm^−1^, representative for the BSA amide I and amide II bonds^22^,^23^, which suggests that the cross-linking process between -CHO in GA and -NH_2_ in BSA may not form special functional groups except CO-NH bonds (peptide bonds). However, one possibility we cannot rule out is that the formation of new chemical bonds (if any) could be too low to be detected in the FTIR spectrum due to the low sensitivity (5%) of the FTIR equipment.

### Mechanical property studies of cross-linked BSA hydrogel

**Figure 6:**
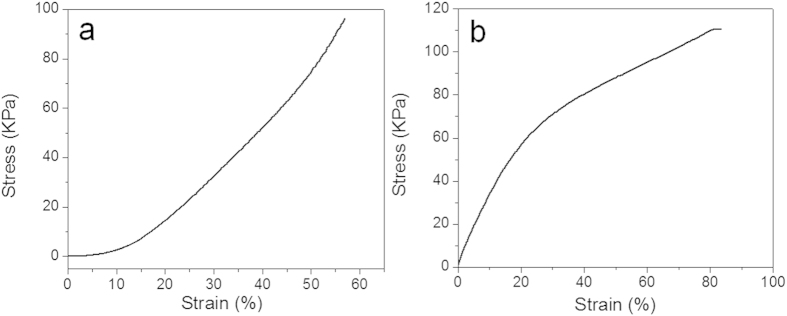
(**a**) Compression test and (**b**) Tensile test results.

As the mechanical integrity of a hydrogel is an important design specification for *in vitro* and *in vivo* applications, both compression and tensile tests have been widely used to examine the mechanical properties of different types of hydrogels^24^. For *in vivo* applications, the hydrogel should be pliable enough to coexist with surrounding tissue but also possess enough structural integrity to withstand intraluminal and subcutaneous pressure from surrounding structures. Therefore, a compression analysis was first carried out (Fig. 6a). To study the compressive performance of the BSA hydrogel, a constant preload force of 0.01 N and ramp force of 0.5 N/min up to 18 N at 37 °C was applied. The stress-strain characteristic of the BSA hydrogel displayed a typical trend for protein hydrogels^25^. The Young’s modulus characterizes the pure elastic domain of a material where the deformation due to applied stress is reversible. However, the stress-strain curve for the BSA hydrogel investigated here is nonliner; hence a global Young’s modulus of compression could not be determined. The experimental result also shows that the hydrogel has been pressed with dimensional changes under this force range and no breaking point of the hydrogels could be observed under pressures up to 18 N. A tensile mechanical measurement was also performed on the BSA hydrogel with a preload force of 0.01 N and ramp force of 0.05 N/min up to 18 N at 37 °C. Figure 6b shows a typical stress-strain curve of the hydrogel during a typical tensile test^25^. The hydrogel length can be elongated as much as 1.8 folds before rupture. Overall, the high molecular weight of BSA protein with extensive cross-linked covalent bonds through GA has led to a hydrogel with high mechanical strength^25^,^26^.

### Cytotoxicity and *in vitro* enzymatic degradation studies of cross-linked BSA hydrogel

Injectable materials are advantageous in that they can be applied to tissue defects with irregular shapes and form tight interfaces with surrounding tissues^27^. As it is difficult to remove the unreacted residues after injection and curing, biocompatibility and biodegrability are two most important factors to evaluate the merits of hydrogels. Thus cytotoxicity was first conducted to evaluate *in vitro* biocompatibility of the BSA hydrogel using a cell-based indirect contact test^27^.

**Figure 7:**
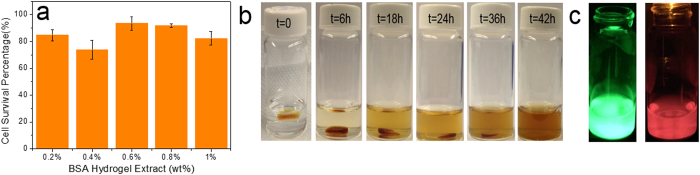
(**a**) Cell survival rate versus BSA hydrogel extract in cytotoxicity test; (**b**) BSA hydrogel digested by proteinase K; (**c**) Fluorescence images of the digested BSA hydrogel excited at a wavelength of λ = 470 nm and λ = 595 nm and collected using a λ = 530 nm optical filter and a λ = 630 nm optical filter, respectively.

Since glutaraldehyde has a certain level of cytotoxicity in the cells, the freeze-drying process was employed to eliminate the unreacted glutaraldehyde and the lyophilized BSA hydrogel was used. While there is some new fluorescent compounds formed during the cross-linking process, Fig. 7a shows that more than 80% of the A549 cells remained alive after exposure to different concentrations of hydrogel extracts in media. These results indicate that the cross-linked BSA hydrogel and newly formed fluorescent compounds are relatively noncytotoxic, which provides solid ground for the latter *in vivo* biocompatibility test.

The *in vitro* biodegradability of the hydrogel was tested by adapting a similar proteinase K method reported by Zhao *et al.*^28^ Proteinase K is a widely used enzyme for protein digestion, because it can hydrolyze the amine bonds and also break ester bonds. In order to track the color changes of the solution during the whole degradation process, the enzyme solution was not refreshed throughout the test. Considering the fact that the activity of the proteinase K gradually decreases over time, the proteinase K concentration used was 1 mg/mL. As shown in Fig. 7b, the cross-linked BSA hydrogel was fully digested after a 42 h incubation at 37 °C. The color of the solution became brownish over time, which can be attributed to the release of the colorful fluorescent compounds accompanying the BSA hydrogel digestion. After the digestion, the solution also exhibited a strong green and red fluorescence (Fig. 7c), indicating that the fluorescent compounds are stable and cannot be damaged during the degradation process. Such a claim was also corroborated by subsequent *in vivo* tests.

### *In vivo* biodegradability study and degradation/diffusion modeling of cross-linked BSA hydrogel

Since *in vitro* biodegradability and biocompatibility studies showed promising results, pilot *in vivo* experiments were carried out using mouse models. Nude mouse is chosen in this study because it has no hair and is convenient to easily track the fluorescence image non-invasively.

**Figure 8:**
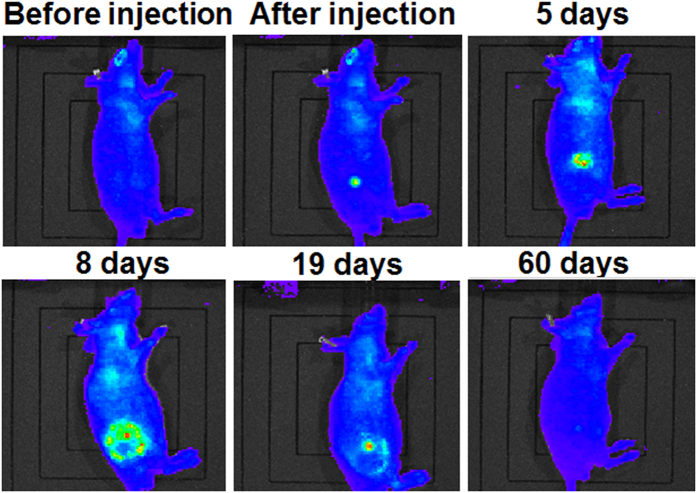
Fluorescence images of the 7-month old mouse with injected BSA hydrogel acquired at different time period.

The cross-linked BSA hydrogels were first subcutaneously injected into two 7-month old Athymic Nude (nu/nu) mice using a modified indwelling needle assembly^29^, and the corresponding fluorescence images at the injection site of the mice were *in-situ* tracked at different time points for 2 months using IVIS Lumina LT system and the corresponding results were presented in Fig. 8, indicating that the cross-linked BSA hydrogel was completely digested *in vivo* in 2 months.

**Figure 9:**
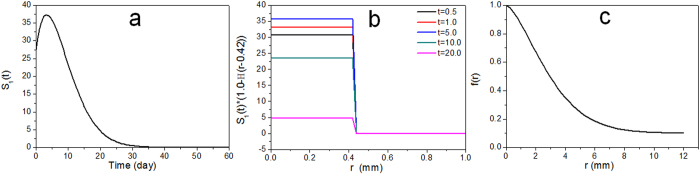
Empirical parameters applied to the mathematical model: (**a**) scaled strength *A* = 500, shape factor *k* = 1.5, scale parameter λ = 10 day, and phase lag T = 1.5 day for the Weibull function, (**b**) source term *S*(*r*, *t*), radius *R* = 0.42 mm for a modified gauge-18 indwelling needle, (**c**) the fitted initial condition *c*(*r*, 0) = 0.1 + 0.8 exp(−0.16*r*^1.5^).

Immediately after subcutaneous injection, strong fluorescence was also observed at the injection site when images were recorded at an emission of 595 nm with an excitation wavelength of 535 nm. Such non-invasive real-time fluorescence tracking allowed for the observation of the fluorescent BSA hydrogel’s *in vivo* degradation process. A sequence of fluorescent images (Fig. 8) shows the radiant fluorescence intensity corresponding to the transient distribution of the fluorophores released from the gel, which was subcutaneously injected. A strong and localized radiant fluorescence intensity was detected immediately after the injection, and the intensity decayed and the fluorescent area broadened. The fluorescence appears to be circularly symmetric with a few patchy spots within this region. The radiant fluorescence intensity remained detectable up to two months when the fluorophores were fully cleared from the injection site. The temporal correlation of the radiant fluorescence and the concentration of the fluorophores can be established using the proposed physics-based model. In order to model the process, an axisymmetric, two-dimensional, and long-time Brownian diffusion of the fluorophores released from the injection point due to the degradation of autofluorescent hydrogel was assumed. The amount of injected hydrogel and the radiant fluorescent intensity were scaled by the maximum observable intensity for convenience. The fluorophore release was mimicked by a source term composed by a product of Weibull and complementary Heaviside functions. The Weibull function describes the growth and attenuation of the fluorescent signal from the hydrogel, while the complementary Heaviside step function determines the spatial domain of the gel. The source function can thus be expressed as


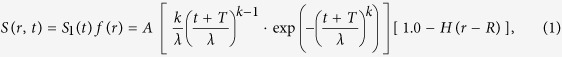


where *r* is radial position, *t* is time, *T* is phase lag to match the initial condition, the constant *A* adjusts the overall strength of the source, *k* and *λ* are the shape factor and scale parameter of the Weibull function, respectively, *R* is the gel radius or the inner radius of injection needle, and *H* is the Heaviside step function. The temporal (*S*_1_) and spatial (*f*) contributions of the source term *S* are shown in Fig. 9. The Weibull function gives a flexible adjustment on the growth and decay of the radiant intensity due to the released fluorescent compound’s diffusion, BSA hydrogel degradation, attenuation or absorption of the fluorophores, etc.

Once the source term was determined, the transient species concentration correlated to the radiant intensity can be described by the diffusion equation:


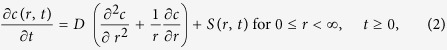


where *D* is an apparent constant diffusivity. We further assume that the initial condition can be formulated by an exponential function with an adjustable power (Fig. 9c):


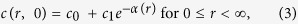


where the constants *c*_0_ and *c*_1_ and the power function *α*(*r*) can be used to fit the observed initial radiant intensity. The corresponding boundary conditions are


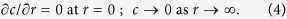


**Figure 10:**
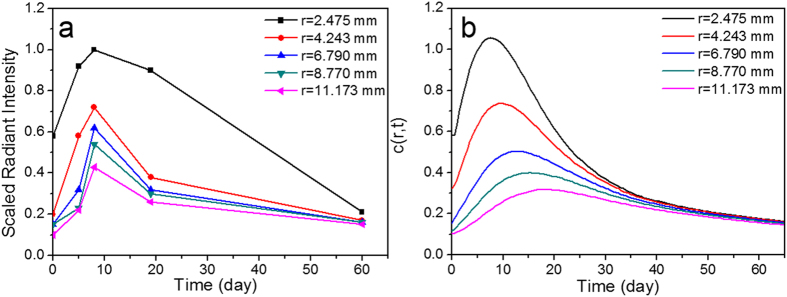
(**a**) Extracted radiant fluorescence intensity (dots) versus time for five various locations at *r* = 2.475, 4.243, 6.790, 8.770, and 11.170 mm. (**b**) The calculated results on the transient distribution of apparent fluorophore concentration at the corresponding locations. The diffusivity *D* is approximately 4.32 mm^2^/day.

The above mathematical model has an analytical solution in an integral form, which can be derived by the Hankel transform of order zero, written as


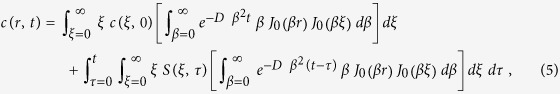


where *J*_0_ is zeroth-order Bessel function, *β* indicates continuous eigenvalues, and *ξ* and *τ* are spatial and temporal dummy indices. The contributions of the initial condition and the source function are given by the first and second integrals. Figures 10a,b shows the comparison of experimental and theoretical results. The experimental data show characteristic diffusion time around 20 days, a diffusive length about 10 mm, and thus it is reasonable to assume the apparent *in vivo* diffusivity as 4.32 mm^2^/day, which is about 5% of the diffusivity of a small molecule in water (~10^–9^ m^2^/s). Several phenomenological parameters are involved in the initial condition and the source term that require further validation, however, apparent diffusivity *D* is the only empirical parameter in this physics-based model that simulates the transient release and redistribution of the fluorophores. Overall the calculated results are very consistent with the radiant fluorescence intensity observed (Fig. 10). The time delay of the growth of the signal was due to the degradation of the hydrogel and the accompanying release of fluorescent compounds, and a further time shift of the peak values at various location is typical for a diffusive wave that propagates outward to a broader region. The calculation very well predicts the characteristic features of the underlying diffusive process. Quantitative consistency may be improved by further *in vitro* and *in vivo* experiments that can statistically validate the empirical parameters we have applied to this model.

**Figure 11:**
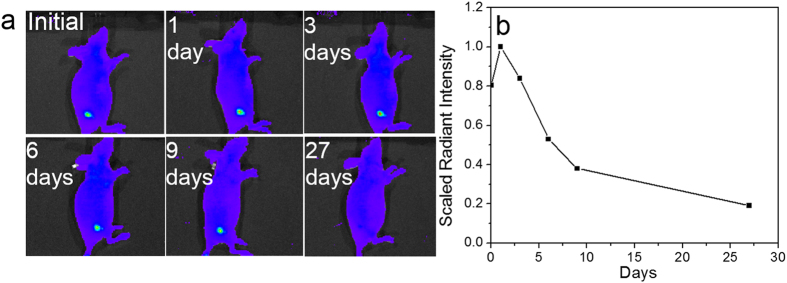
(**a**) Fluorescence images of the 2-month old mouse with injected BSA hydrogel acquired at different time period. (**b**) Extracted radiant fluorescence intensity (dots) versus time.

As the age of mouse may also play the role in the *in vivo* degradtation of injected hydrogel as well as the removal of released fluorophores, similar experiment was preliminarily carried out using younger mice(2-month old). As shown in Fig. 11a, the younger mouse exhibits a similar degradation trend as the aged one, however, it is obvious that the degradation process is much faster than the aged mice - From the third day post-injection, fluorescence intensity has begun to decline, and at 27 days, the fluorescence signal disappears. They also exhibited different diffusion patterns in terms of fluorescent signal. Such difference can be presumably attributed to the variance of metabolism efficiency in young and old mouse. Further *in vivo* studies need to be conducted to clarify the degradation variation of the hydrogel in mice. Fig. 11b shows the fluorescence intensity change along with the degradation process, which initially increases and then diminishes. The histology study was also carried out for the young nude mouse with hydrogel injection and the corresponding result was presented in Figure S3. One can see that all of tissues are normal and no inflammation appears, which is similar as the histology result of the old one discussed in the next section.

These results implied that glutaraldehyde cross-linked protein hydrogel is a promising autofluorescent biodegradable material and can therefore serve as an excellent platform to investigate the *in vivo* degradation of biomaterials without using any fluorophore labels and to develop an appropriate mathematical model to understand and/or predict the degradation pattern of the implanted biomaterials.

### *In vivo* biocompatibility study of cross-linked BSA hydrogel

**Figure 12:**
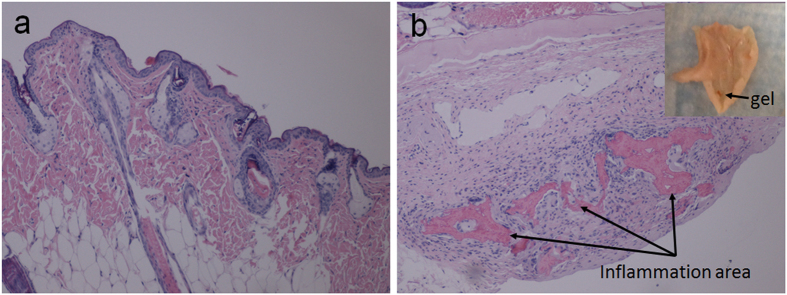
(**a**) skin tissue images in which hyperkeratosis was shown on the surface of epidermis (100×); **(b)** skin tissue focus on subcutaneous area. Inset shows the BSA hydrogel residue in th imjection site (40×) (20 days post injection).

A *in vivo* biocompatibility study was also conducted. The tissues from mice were collected separately for histology analysis. For Mouse 271, the hydrogel was injected 20 days prior to tissue collection and it was not fully degraded (the black arrow indicates the location of the BSA hydrogel residue in Fig. 12b). In Figure 12, there was a mild hyperkeratosis on the surface of the epidermis that extended into a few hair follicles. A few hair follicles contained fragments of immature hair shafts. There was one focus of a few neutrophils with a few karyorrhectic nuclei and macrophages that had infiltrated into the subcutis. In the subcutaneous region, the injection area was surrounded by macrophages, some giant cells, and proliferating connective tissue infiltrated by a few neutrophils and eosinophils. The connective tissue was forming a capsule walling off the inflammation area. Up to 20 days, the autofluorescent BSA hydrogel was not fully digested and an inflammatory response was observed unsurprisingly due to the needle invasion, however, it could disappear after the hydrogel was fully digested, which was demonstrated in Mouse 267.

**Figure 13:**
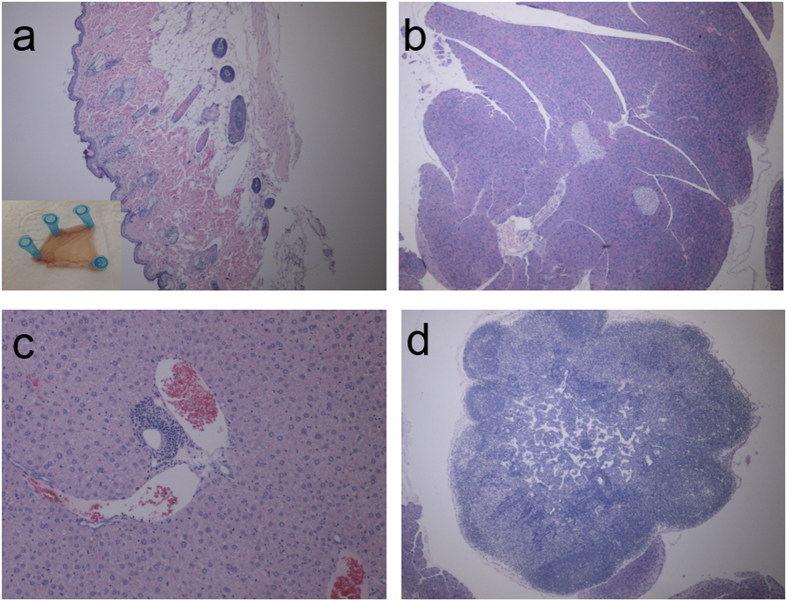
The histology study. (**a**) Skin tissue images (Inset shows the skin around the injection site) (40×); (**b**) Pancreas tissue image (40×); (**c**) Liver tissue images (100×); (**d**) Lymph node tissue images (40×).

Mouse 267, whose BSA hydrogel had been fully degraded in 2 months, was also subject to the histology study, as shown in Fig. 13. For skin, there was a mild hyperkeratosis extending into a few hair follicles. Otherwise there were no significant lesions (Fig. 13a). For pancreas, there is no significant lessions (Fig. 13b). For liver, there were a few foci of accumulations of lymphocytes and plasma cells in portal areas surrounding bile ducts. Otherwise the liver had no significant lesions (Fig. 13c). No significant lesions were observed in lymph nodes (Fig. 13d). The tissues examined histologically were within normal limits in this mouse. This histology study further demonstrated *in vivo* biocompatibility of the as-developed autofluorescent BSA hydrogel.

## Discussion

In our study, it has been demonstrated that glutaraldehyde cross-linked BSA hydrogel is highly biocompatible according to the *in vitro* cytotoxicity test and *in vivo* biocompatibility experiment. The BSA hydrogel exhibits strong green and red fluorescence *in vitro* and *in vivo* which can be excited over a wide range of wavelengths. According to the results from UV-vis and fluorescence spectra, it suggests that three classes of fluorescent compounds might be formed in BSA hydrogel after using GA as the cross-linker. In addition, the surface morphology of the BSA hydrogel exhibited a highly porous structure. The biodegradability and *in vivo* degradation of the BSA hydrogel was tracked and modeled without using any additional fluorphores, due to its unique autofluorescence, which broadens its suitability in a wide spectrum of biomedical applications. Since both the fluorescence of the cross-linked BSA hydrogel and the fluorescent compounds in the digested hydrogel solution are highly stable over months on the bench, the identification of the newly formed fluorescent compounds in GA cross-linked BSA hydrogel became of particular interest. In this regard, LC-MS was further employed to analyze the proteinase K digested sample as trypsin could not fully digest the cross-linked BSA hydrogel. However, the LC-MS results (Figure S1) could not provide confirmative information due to the complexity and randomness of BSA cross-linking process with GA. Recently, there are few studies about autofluorescence generation in cell fixation and some peptides by glutaraldehyde^30^ and autofluorescence of glutaraldehyde cross-linked chitosan nanoparticles^31^. The secondary amine and ethylenediamine in the molecule have been proposed to contribute to the fluorogenic mechanism^30^. Also, in glutaraldehyde solutions, α,β-unsaturated aldehyde polymer can be formed, which leads to the C=C double bond existed in the system, accompanied by the C=N double bonds from schiff’s base. Thus, it’s highly possible that the fluorescence observed in the GA cross-linked BSA hydrogel are attributed to the electronic transitions such as π-π* transition of C=C bond and n-π* trasnsition of C=N bond^31^. More complicatedly, the protein’s secondary and/or tertiary structure may also have significant influence on the synthesis of the fluorescent compounds and the fluorescence observed might be attributed to the synergistic effect of several amino acids in close proximity, for example, similar to other green or red fluorescent proteins. Moreover, the charge surrounding the flurophores in BSA hydrogel is another potential factor to affect the fluorescence. More efforts are required in order to identify the chemical structure of such stable fluorescent compounds and thus creat them using organic synthesis for other potential applications.

As the fluorescence technology represents one of the most promising non-invasive approaches to monitor physiological parameter changes and to track the *in vivo* degradation of biomaterials, the newly synthesized autofluorescent BSA hydrogel with high biocompatibility and biodegradability exhibits high value in a variety of biomedical applications ranging from biosensing to drug delivery and tissue engineering. As for the *in vivo* biodegradation modeling, the fluorescence intensity first enhanced and then decayed along the time. Such observation might be explained as follows: the autofluorescence of the concentrated BSA hydrogel is initially self-quenched to certain degree due to the fact that the excitation photons are limited in a small area, and as it degrades, the fluorophores diffuse out along the time. Consequently, the increase of the fluorescence intensity was observed. The released fluorophores have the chance to diffuse out and reach other areas instead of immediate clearance by the immune response, which may be attributed to the lack of functional T-cell in nude mice. Over a course of couples of weeks, the released fluorophores gradually disappeared through circulation system. More studies are required in order to prove the biological meaning of the developed mathematical model.

This facile strategy to prepare unique autofluorescent BSA hydrogels will open a new venue to prepare novel biocompatible and biodegradable fluorescent protein hydrogels, which could significantly expand the scope of protein hydrogel in biomedical applications.

## Methods

### Cross-linked BSA or HSA Hydrogel

In this study, BSA and glutaraldehyde solution was mixed quickly with the final BSA and GA concentrations of 20% and 1%, respectively. After tens of seconds, autofluorescent BSA hydrogel was prepared. The gelation time was determined using an reported inverted tube method^21^. BSA concentration varying from 10 ~ 30% is also able to be cross-linked by 1% glutaraldehyde to form the unique autofluorescent hydrogel. In addition, human serum albumin (HSA) can be used to replace BSA and form autofluorescent hydrogel too.

### Freeze-Dry BSA Hydrogel

The BSA hydrogel was prepared in a small tuber and hung using a stainless steel holder in order to freeze the hydrogel in liquid nitrogen. Then the freeze-dry flask containing the stainless steel holder was placed on a vacuum lyophilization equipment for an appropriate time to remove water in hydrogel. The time for lyophilization was determined by the volume of the hydrogel sample.

### Characterization of the BSA hydrogel

In the UV-vis study, BSA hydrogel was prepared in a 3 mL cuvette, and the BSA solution was prepared in a quartz cuvette as a comparison. The UV-vis spectra were collected from 200 nm to 800 nm using a Varian Cary 50 Scan UV-Visible Spectrophotometer.

To obtain the fluorescent images, two sets of filters were employed, including Ex/Em = 470 nm/530 nm and Ex/Em = 595 nm/630 nm. The Excitation filter was placed in front of the LED light to excite the hydrogel with the specific wavelength and the Emission filter was attached on the camera to record the emission image of the BSA hydrogel.

Varian Cary Eclipse Fluorescence Spectrophotometer was used in the fluorescence spectra study. BSA hydrogel was prepared in a 3 mL cuvette. In the emission scan, excitation wavelength was set from 370 nm to 600 nm with a 10 nm increments in each scan and emission wavelength was set from a wavelength slightly above the excitation wavelength to 800 nm. In the excitation scan, emission wavelength was set at 510 nm, 550 nm, and 602 nm, respectively, and the excitation scanned from an appropriate wavelength to a wavelength slightly less than the emission wavelength. The synchronous scan was conducted with the start wavelength of 400 nm, the stop wavelength of 700 nm and a Delta of 20 nm.

Surface morphology of the prepared hydrogel was studied using JEOL 6335 Field Emission Scanning Electron Microscope (FESEM) operated at an accelerating voltage of 10 kV and 12 μA. BSA hydrogel was prepared and flash-frozen in liquid nitrogen and lyophilized. Prior to imaging, the sample cross-sections were cut by razor blade and mounted on double-sided carbon tape stuck on SEM stubs. Further, the samples were sputtered coated with Au/Pd alloy for improved conductivity. Attenuated total reflectance (ATR) infrared spectra of dry BSA hydrogel and BSA powder were obtained with a Thermo Nicolet IR 560 system, using a Zn-Se ATR accessory (Thermo Electron Corporation, PA). Each sample was placed against the ATR element and the spectra were collected in the range of 500–4000 cm^–1^ using 128 scans at a resolution of 4 cm^–1^. After acquisition, the IR spectra were baseline corrected for carbon dioxide peak at approximately 2750 cm^–1^.

Mechanical analysis of the hydrogel samples were performed on TA instruments Q800 under controlled force (compression and tensile mode). A pair of compression disc clamps of 15 mm was used on uniform hydrogel sample. Constant preload force of 0.01 N and ramp force of 0.5 N/min was applied up to 18 N at 37 °C.

### *In vitro* cytotoxicity test

BSA hydrogel was prepared as the previous procedure and then 100 mg freeze-dried hydrogel was soaked in 5 mL (2%) Dulbecco’s Modified Eagle Medium (DMEM) and incubated at 37 °C for 24 h. Concurrently, 5,000 A549 cells were seeded per well to a 96-well plate with an overnight incubation. The cells were treated with 0, 0.2, 0.4, 0.6, 0.8 and 1% hydrogel extract diluted with DMEM. After a 24h-incubation at 37C, the samples were removed and replaced with the fresh medium (100 μL per well). Finally 10 μL of CCK-8 solution was loaded to each well and the absorption at 450 nm on a microplate reader was recorded after 1 hour incubation. The cell survival percentage was calculated with following equation:


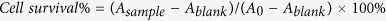


### *In vitro* enzymatic biodegradation test

*In vitro* degradation test was conducted in a small glass vial (Fig. 7b) containing a 300 μL BSA hydrogel and 5 mL of PBS buffer (pH 7.4 10 mM) with a relative higher proteinase K concentration at 1 mg/mL. The optical pictures were recorded at different time intervals (0 h, 6 h, 18 h, 24 h, 36 h, and 42 h).

### Pilot *In vivo* biodegradability and biocompatibility study

All studies were performed with a University of Connecticut Institutional Animal Care and Use Committee approved protocol and the methods were carried out in accordance with the approved guidelines. Freeze-dried sample was autoclaved first, and then soaked in the sterilized PBS buffer to absorb water and swell back to form hydrogel. Before injection of BSA hydrogel, the mouse was anesthetized using isoflurane and the implantation area was cleaned with an alcohol swab. Then the hydrogel prepard in advance was subcutaneously injected in two 7-month old Athymic Nude mice (Mouse 267 and Mouse 271) and a 2-month old Athymic Nude mice (Mouse 189) using an modified 18-gauge indwelling needle assembly. The whole needle assembly was then removed and the area was wiped again with an alcohol swab to clean the area before returning the mouse back to the cage. The area was then monitored for inflammation and fluorescent images continuously. After BSA hydrogel injection, the 7-month old Mouse 271 and Mouse 267 were sacrified at 20 days and 2 months, respectively, while the 2-month old Mouse 189 was sacrificed at 27 days, to conduct the histology analysis.

### LC-MS

After proteinase K digestion, hydrogel samples were analyzed by reverse-phase HPLC-ESI-MS/MS using an Shimadzu HPLC system coupled to a hybrid quadrupole time-of-flight QSTAR Elite mass spectrometer with a TurboSpray Ionization Source (AB SCIEX, USA). Briefly, 60 picomole digested sample were loaded onto C18 column (Inertsil ® ODS-3, 2.1 × 150 mm, 3 μm particle size, 100 Å pore size, GL Science Inc., Japan) and eluted at a flow rate of 100 μL/min using the following gradient: 0–4 min 5% solvent B, 4–40 min 5–40% B, 40–43 min 40–80% B, 43–48 min 80% B, 48–49 min 80-5% B, 49-60 min 5% B, with a total runtime of 60 min. Mobile phases are 0.1% formic acid (v/v) in water (A) and 0.1% formic acid (v/v) in acetonitrile (B). Mass spectra were recorded in positive ion mode. For collision induced dissociation tandem mass spectrometry (CID-MS/MS), the precursor ions were fragmented in a collision cell using nitrogen as the collision gas. Advanced information dependent acquisition (IDA) was used for MS/MS collection to obtain MS/MS spectra for the 2 most abundant parent ions following each survey scan in m/z range from 300 to 1600. Dynamic exclusion duration was set as 30 sec.

## Additional Information

**How to cite this article**: Ma, X. *et al.* A Biocompatible and Biodegradable Protein Hydrogel with Green and Red Autofluorescence: Preparation, Characterization and *In Vivo* Biodegradation Tracking and Modeling. *Sci. Rep.* **6**, 19370; doi: 10.1038/srep19370 (2016).

## Supplementary Material

Supplementary Information

## References

[b1] Shen W., Lammertink R. G. H., Sakata J. K., Kornfield J. A. & Tirrell D. A. Assembly of an artificial protein hydrogel through leucine zipper aggregation and disulfide bond formation. Macromolecules 38, 3909–3916 (2005).

[b2] Peppas N. A., Hilt J. Z., Khademhosseini A. & Langer R. Hydrogels in biology and medicine: From molecular principles to bionanotechnology. Adv Mater 18, 1345–1360 (2006).

[b3] Drury J. L. & Mooney D. J. Hydrogels for tissue engineering: scaffold design variables and applications. Biomaterials 24, 4337–4351 (2003). 12922147 10.1016/s0142-9612(03)00340-5

[b4] Lee K. Y. & Mooney D. J. Hydrogels for tissue engineering. Chem Rev 101, 1869–1879 (2001). 11710233 10.1021/cr000108x

[b5] Park J. H. & Bae Y. H. Hydrogels based on poly (ethylene oxide) and poly (tetramethylene oxide) or poly (dimethyl siloxane): synthesis, characterization, *in vitro* protein adsorption and platelet adhesion. Biomaterials 23, 1797–1808 (2002). 11950050 10.1016/s0142-9612(01)00306-4

[b6] Graiver D., Durall R. & Okada T. Surface morphology and friction coefficient of various types of Foley catheter. Biomaterials 14, 465–469 (1993). 8507794 10.1016/0142-9612(93)90150-z

[b7] Risbud M. V. & Bhonde R. R. Polyacrylamide-chitosan hydrogels: *in vitro* biocompatibility and sustained antibiotic release studies. Drug Delivery 7, 69–75 (2000). 10892406 10.1080/107175400266623

[b8] Censi R., Martino P. D., Vermonden T. & Hennink W. E. Hydrogels for protein delivery in tissue engineering. Journal of Controlled Release 161, 680–692 (2012). 22421425 10.1016/j.jconrel.2012.03.002

[b9] Sun X., Ma X., Kumar C. V. & Lei Y. Protein-based sensitive, selective and rapid fluorescence detection of picric acid in aqueous media. Analytical Methods 6, 8464–8468 (2014).

[b10] Matsushita S. *et al.* Functional analysis of recombinant human serum albumin domains for pharmaceutical applications. Pharmaceutical research 21, 1924–1932 (2004). 15553241 10.1023/b:pham.0000045248.03337.0e

[b11] Migneault I., Dartiguenave C., Bertrand M. J. & Waldron K. C. Glutaraldehyde: behavior in aqueous solution, reaction with proteins, and application to enzyme crosslinking. Biotechniques 37, 790–806 (2004). 15560135 10.2144/04375RV01

[b12] Nimni M. E., Cheung D., Strates B., Kodama M. & Sheikh K. Chemically modified collagen: a natural biomaterial for tissue replacement. Journal of biomedical materials research 21, 741–771 (1987). 3036880 10.1002/jbm.820210606

[b13] Habeeb A. & Hiramoto R. Reaction of proteins with glutaraldehyde. Archives of biochemistry and biophysics 126, 16–26 (1968). 4174905 10.1016/0003-9861(68)90554-7

[b14] Jun J. Y., Nguyen H. H., Chun H. S., Kang B.-C. & Ko S. Preparation of size-controlled bovine serum albumin (BSA) nanoparticles by a modified desolvation method. Food Chemistry 127, 1892–1898 (2011).

[b15] Artzi N. *et al.* *In vivo* and *in vitro* tracking of erosion in biodegradable materials using non-invasive fluorescence imaging. Nature materials 10, 704–709 (2011). 21857678 10.1038/nmat3095 PMC3160718

[b16] Wang W. *et al.* Real-time and non-invasive fluorescence tracking of *in vivo* degradation of the thermosensitive PEGlyated polyester hydrogel. Journal of Materials Chemistry B 2, 4185–4192 (2014). 10.1039/c4tb00275j 32261752

[b17] Heo Y. J., Shibata H., Okitsu T., Kawanishi T. & Takeuchi S. Long-term *in vivo* glucose monitoring using fluorescent hydrogel fibers. Proceedings of the National Academy of Sciences 108, 13399–13403 (2011). 10.1073/pnas.1104954108 PMC3158145 21808049

[b18] Suri J. T., Cordes D. B., Cappuccio F. E. Wessling R. A. & Singaram B. Continuous glucose sensing with a fluorescent thin‐film hydrogel. Angewandte Chemie International Edition 42, 5857–5859 (2003). 10.1002/anie.200352405 14673918

[b19] Bhuniya S. & HyeanáKim B. An insulin-sensing sugar-based fluorescent hydrogel. Chemical communications 17, 1842–1844 (2006). 10.1039/b516632b 16622502

[b20] Gong Y., Gao M., Wang D. & Möhwald H. Incorporating fluorescent CdTe nanocrystals into a hydrogel via hydrogen bonding: toward fluorescent microspheres with temperature-responsive properties. Chemistry of materials 17, 2648–2653 (2005).

[b21] Wu Y., Wang L., Guo B. & Ma P. X. Injectable biodegradable hydrogels and microgels based on methacrylated poly (ethylene glycol)-co-poly (glycerol sebacate) multi-block copolymers: synthesis, characterization, and cell encapsulation. Journal of Materials Chemistry B 2, 3674–3685 (2014). 10.1039/c3tb21716g 32263804

[b22] Lad M. D., Birembaut F., Matthew J. M., Frazier R. A. & Green R. J. The adsorbed conformation of globular proteins at the air/water interface. Physical Chemistry Chemical Physics 8, 2179–2186 (2006). 16751876 10.1039/b515934b

[b23] Subia B. & Kundu S. Drug loading and release on tumor cells using silk fibroin–albumin nanoparticles as carriers. Nanotechnology 24, 035103; doi: 10.1088/0957-4484/24/3/035103 (2013). 23262833

[b24] Svensson A. *et al.* Bacterial cellulose as a potential scaffold for tissue engineering of cartilage. Biomaterials 26, 419–431 (2005). 15275816 10.1016/j.biomaterials.2004.02.049

[b25] Dai X. *et al.* A Mechanically Strong, Highly Stable, Thermoplastic, and self‐healable supramolecular polymer hydrogel. Advanced Materials 27, 3566–3571(2015). 25946310 10.1002/adma.201500534

[b26] Sun J.-Y. *et al.* Highly stretchable and tough hydrogels. Nature 489, 133–136 (2012). 22955625 10.1038/nature11409 PMC3642868

[b27] Shin H., Temenoff J. S. & Mikos A. G. *In vitro* cytotoxicity of unsaturated oligo [poly (ethylene glycol) fumarate] macromers and their cross-linked hydrogels. Biomacromolecules 4, 552–560 (2003). 12741769 10.1021/bm020121m

[b28] Zhao C. *et al.* Synthesis of biodegradable thermo-and pH-responsive hydrogels for controlled drug release. Polymer 50, 4308–4316 (2009).

[b29] Shibata H. *et al.* Injectable hydrogel microbeads for fluorescence-based *in vivo* continuous glucose monitoring. Proceedings of the National Academy of Sciences 107, 17894–17898 (2010). 10.1073/pnas.1006911107 PMC2964191 20921374

[b30] Lee K., Choi S., Yang C., Wu H.-C. & Yu J. Autofluorescence generation and elimination: a lesson from glutaraldehyde. Chemical Communications 49, 3028–3030 (2013). 23462727 10.1039/c3cc40799c

[b31] Wei W. *et al.* Preparation and application of novel microspheres possessing autofluorescent properties. Advanced Functional Materials 17, 3153–3158 (2007).

