# Supplementary material for: A Biocompatible and Biodegradable Protein Hydrogel with Green and Red Autofluorescence: Preparation, Characterization and In Vivo Biodegradation Tracking and Modeling
Source: Sci Rep. 2016 Jan 27;6:19370. doi: 10.1038/srep19370 (PMC4728389; doi:10.1038/srep19370)
Supplement: Supplementary Information [file srep19370-s1.pdf]

Supplement Materials

A Biocompatible and Biodegradable Protein Hydrogel with Green and Red Autofluorescence: Preparation, Characterization and *In Vivo* Biodegradation Tracking and Modeling

Xiaoyu Ma[1], Xiancheng Sun[2], Derek Hargrove[3], Jun Chen[1], Donghui Song[3], Qiuchen Dong[1], Xiuling Lu[3], Tai-Hsi Fan[4], Youjun Fu[5], Yu Lei[1,2*]

[1]Department of Biomedical Engineering, University of Connecticut, Storrs, CT 06269, USA
[2]Department of Chemical and Biomolecular Engineering, University of Connecticut, Storrs, CT 06269, USA
[3]Department of Pharmaceutical Sciences, University of Connecticut, Storrs, CT 06269, USA
[4]Department of Mechanical Engineering, University of Connecticut, Storrs, CT 06269, USA
[5]Department of Chemistry, University of Connecticut, Storrs, CT 06269, USA

* To whom correspondence should be addressed:
Email: ylei@engr.uconn.edu

Tel: 1-860-486-4554 Fax : 1-860-486-2959

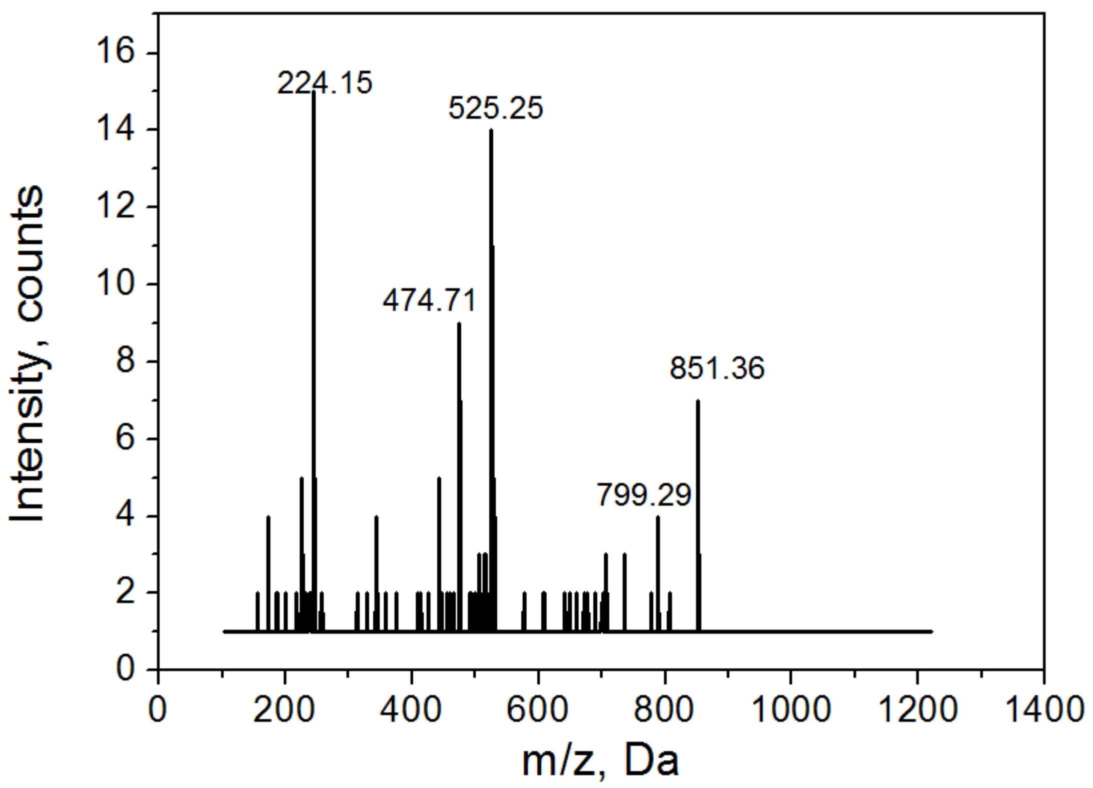

Figure S1| LC-MS result for GA cross-linked BSA hydrogel digested by Proteinase K.

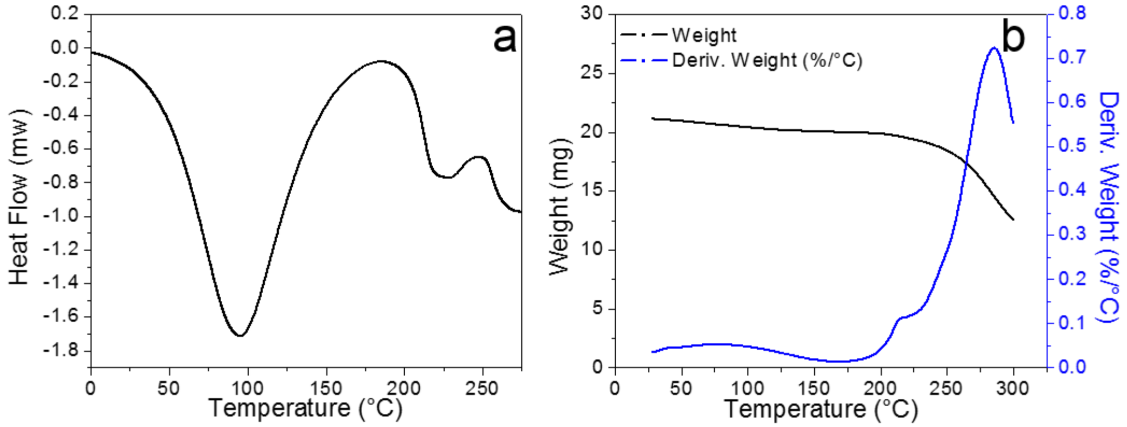

Figure S2| Thermal stability results. (a) DSC thermogram of the cross-linked BSA hydrogel; (b) TGA result of the cross-linked BSA hydrogel.

Differential Scanning Calorimetry (DSC) and Thermogravimetric Analysis (TGA) techniques were employed to study the thermal properties of the cross-linked BSA hydrogel. Unlike BSA particle whose melting point is ~68 °C, the DSC data of the as-prepared cross-linked BSA hydrogel shows a big peak centered at ~95 °C (Fig. S2a), which can be attributed to the fact that

chemical crosslinking of BSA increases the melting point of the hydrogel. TGA study indicates that the as-prepared hydrogel starts to decompose from ~180 °C (Fig. S2b).

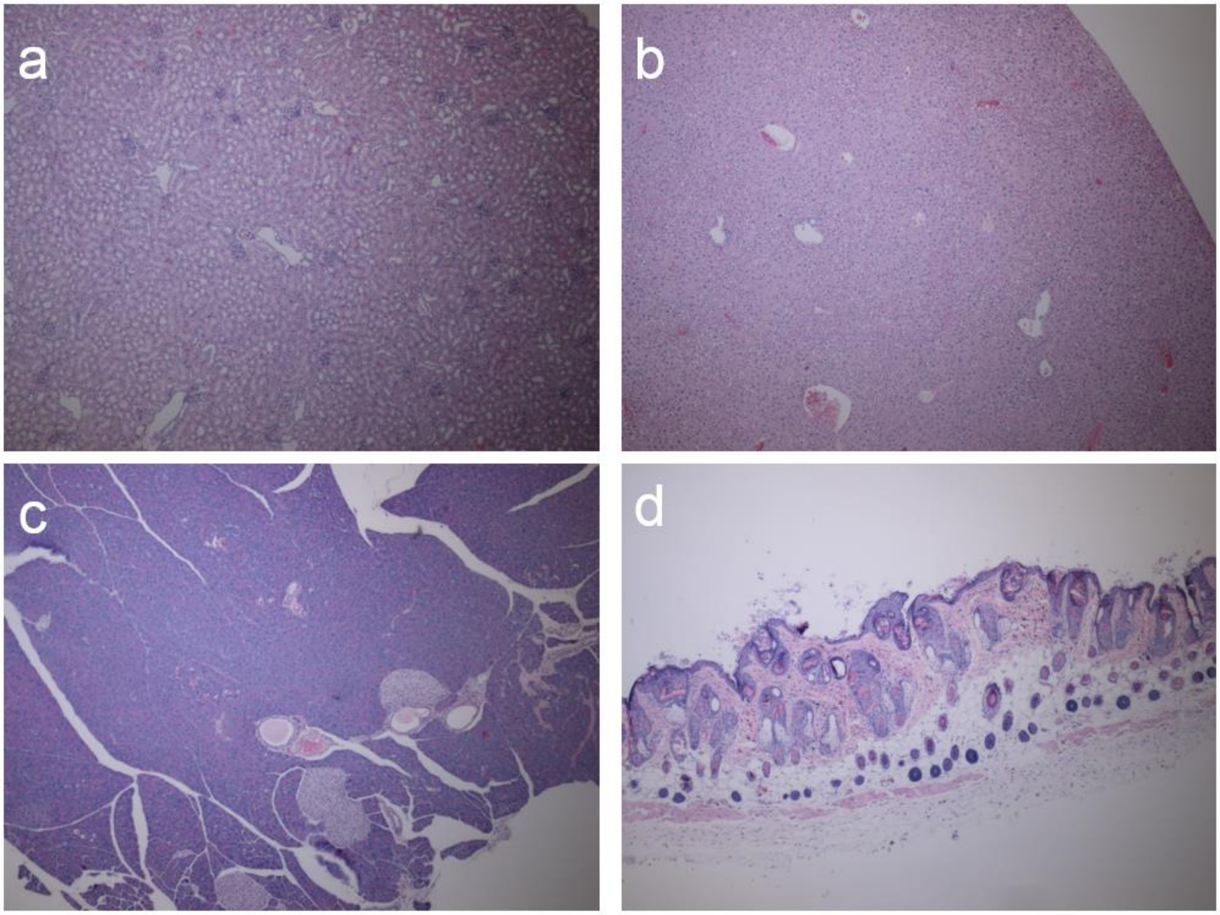

Figure S3| Histology study. (a) Kidney tissue images (40×); (b) Liver tissue image (40×); (c) Pancreas tissue image (40×); (d) Skin tissue image (40×).
